# Overexpression of the *crp* gene promotes biofilm formation and increases antibiotic resistance in bovine-derived *Klebsiella pneumoniae*

**DOI:** 10.3389/fmicb.2026.1766955

**Published:** 2026-01-26

**Authors:** Ying Zhang, Jiancheng Qi, Li Gu, Sirun Yi, Yani Liu, Keyue Zhang, Linqi Guo, Zhicai Zuo

**Affiliations:** College of Veterinary Medicine, Sichuan Agricultural University, Chengdu, Sichuan, China

**Keywords:** antibiotic resistance, biofilm formation, cAMP receptor protein, co-trimoxazole, *Klebsiella pneumoniae*

## Abstract

**Background:**

The multidrug resistance of bovine-derived *Klebsiella pneumoniae* is a significant concern, with biofilm formation serving as a major factor in the escalation of antibiotic resistance. The function of cAMP receptor protein (CRP), which is encoded by the *crp* gene and acts as a central regulator of environmental sensing and virulence, remains unclear in pathogenic strains derived from livestock.

**Purpose:**

This study aims to investigate the influence of CRP overexpression on biofilm formation and antibiotic resistancein bovine-derived *Klebsiella pneumoniae*, with a particular focus on its effect against cotrimoxazole.

**Methods:**

Recombinant strains with constitutive (P*kan*) and inducible (P*tac*) promoter-driven CRP overexpression were constructed using molecular cloning. Gene and protein expression were validated using RT-qPCR and immunoblotting analyses. Biofilm formation was quantified by crystal violet staining, antibiotic susceptibility to 23 agents was assessed using the Kirby-Bauer disk diffusion method, and metabolic burden was evaluated through growth curve analysis.

**Result:**

The CRP-overexpressing strain (KAN group) showed a 2.9-fold increase in CRP protein expression (*p* < 0.01) and a significant enhancement in biofilm formation (*p* < 0.0001), without significant impact on bacterial growth. Notably, a reversal in antibiotic susceptibility was observed: while the wild-type strain was sensitive to cotrimoxazole (inhibition zone: 22 mm), the CRP-overexpressing strain displayed complete resistance (inhibition zone: 7 mm).

**Conclusion:**

Overexpression of CRP protein promotes biofilm formation and confers resistance to cotrimoxazole in bovine-derived *Klebsiella pneumonia*, indicating that CRP-mediated biofilm formation might be a key mechanism driving the observed cotrimoxazole resistance in this strain.

## Introduction

*Klebsiella pneumoniae* (KP) is a significant zoonotic pathogen that poses a serious threat to the beef cattle industry. It is associated with respiratory infections, sepsis, and other diseases, leading to clinical symptoms such as coughing, high fever, and increased mortality, which ultimately lead to substantial economic losses (Wang et al., 2020). Recent epidemiological studies have demonstrated the widespread prevalence of KP among beef cattle in China. For instance, KP accounted for 19.7% of respiratory isolates in Chongqing (Lin et al., 2015) and was detected in 15.6% of diseased cattle in Shenyang (Tang, 2023). Notably, multidrug resistance (MDR) in bovine-derived KB has received growing attention. These KP isolates frequently carry multiple resistance genes, including *shv*, *ctx-M*, and *sul2*, and commonly exhibit high resistance rates to antibiotics such as β-lactams and aminoglycosides, often exceeding 80% (Lin et al., 2015; Tang, 2023; Deng and Luo, 1997). The rise in antibiotic resistance undermines treatment efficacy, complicates recovery, and exacerbates the impact of KP infections in beef cattle. However, the mechanisms underlying MDR in KP remain poorly understood.

A biofilm is a structured ecosystem formed by bacteria that adhere to biotic or abiotic surfaces and secrete extracellular polysaccharides (EPS), proteins, and lipids to encase the microbial community (Ma et al., 2013). In recent decades, biofilm formation has been closely linked to the pathogenicity and antibiotic resistances of KP. Clinical studies have also shown that biofilm formation in carbapenem-resistant *Klebsiella pneumoniae* significantly increases patient mortality risk by 3.2-fold (Di Domenico et al., 2020). Biofilms enhance antibiotic resistance through three primary mechanisms: extracellular matrix act as a physical barrier, significantly reducing antibiotic penetration (Rahdar et al., 2019); the high bacterial density within biofilms (>10^8^ colony-forming units per cubic centimeter, CFU/cm^3^) shortens plasmid transfer distances (Lewis, 2008) and promotes horizontal gene transfer due to increased spatial proximity (Devanga Ragupathi et al., 2020; Lazăr and Chifiriuc, 2010); and the biofilm microenvironment induces the overexpression of efflux pump genes (such as *acrA* and *emrB*), establishing an active efflux defense network (Tang et al., 2020). Although recent studies have demonstrated that biofilm formation is a complex, multistage process rigorously regulated by molecular mechanisms such as type III pili, capsular polysaccharides, quorum sensing, and efflux pumps (Balestrino et al., 2008; Tan et al., 2015; Peng et al., 2018; Chen et al., 2020; Vuotto et al., 2017), the specific regulatory mechanisms responsible for biofilm-associated drug resistance remain largely unclear.

As a global transcriptional regulator, the cyclic AMP receptor protein (CRP) is widely conserved in bacteria and functions as a homodimer. Each subunit comprises two domains: an N-terminal domain responsible for cAMP binding and dimerization, and a C-terminal domain that mediates specific DNA recognition via a helix-turn-helix motif. Upon binding cAMP, CRP undergoes an allosteric conformational change, enabling high-affinity interaction with target DNA sequences and subsequent activation of gene transcription (Fic et al., 2009). Traditionally studied in *Escherichia coli* as a canonical transcription factor regulating catabolite-sensitive genes, recent evidence suggests CRP can also participate in post-translational regulatory networks, such as biofilm maintenance, through direct protein–protein interactions with other signaling effectors (Liu et al., 2022). Given its multifaceted regulatory potential, a critical question arises: how is CRP’s function specifically deployed in *Klebsiella pneumoniae* to influence pathogenesis? In KP, CRP also acts as a master regulator of virulence and adaptation. For instance, CRP positively regulates biofilm formationas, as *crp* gene knockout can reduce biofilm biomass by more than 10-fold reduction, likely through modulating fimbriae production. Additionally, Yang et al. demonstrated that CRP directly binds to the promoter region of the *kfuABC* operon and represses its transcription, suggesting that CRP may also negatively regulate capsule-related genes, such as those involved in iron acquisition (*kfuABC*) and capsular polysaccharide synthesis (Lei and Tan, 2014; Yang, 2014). Furthermore, it was reported to positively regulates the siderophore gene *entC*, thereby dynamically balancing bacterial colonization and resource competition (Yang, 2014; Bi and Du, 2020). Despite these pivotal roles, the precise mechanisms by which CRP coordinates biofilm formation and biofilm-associated antibiotic resistance in KP remain poorly understood.

Therefore, to directly assess the phenotypic consequence of elevated CRP, this study constructed CRP-overexpressing strains of a bovine-derived *Klebsiella pneumoniae* isolate. We systematically compared the wild-type and overexpression strains for their growth kinetics, biofilm-forming capacity, and susceptibility profiles to 23 clinically relevant antibiotics. A key finding was the specific reversal of susceptibility to cotrimoxazole upon CRP overexpression. This work establishes a causal link between CRP levels, enhanced biofilm formation, and specific antibiotic resistance, providing a foundation and critical phenotypic context for future investigations into the underlying molecular mechanisms.

## Materials and methods

### Strains and reagents

The KP-L strain used in this study was isolated in 2018 from the lung of a beef cattle that died of severe pneumonia on a farm in Sichuan Province, China. It was identified as *Klebsiella pneumoniae* and stored as part of our laboratory’s clinical isolate collection. This strain exhibits a multidrug-resistant profile (as detailed in Results) and was selected for its clinical relevance in studying bovine respiratory disease. *Escherichia coli* DH5α competent cells (DH5α) and the kanamycin-resistant plasmid pET28a(+) were purchased from Beijing Solarbio Science & Technology Co., Ltd. (Beijing, China). Since the T7 promoter of pET28a(+) is essentially silent in KP and unsuitable for gene expression in KP (Ma et al., 2010), constitutive promoters P*kan* and P*tac*, both reported to be effective and successfully used for heterologous expression in KP (Ma et al., 2013), were selected to replace the T7 promoter in this study. Taq DNA polymerase was obtained from Nanjing Vazyme Biotech Co., Ltd. (Nanjing, China), and restriction enzymes and DNA ligase were acquired from Takara Biomedical Technology Co., Ltd. (Beijing, China). Plasmids miniprep kits, PCR product purification kits, bacterial genomic DNA extraction kits, and RNA extraction kits were sourced from TIANGEN Biotech Co., Ltd. (Beijing, China). DL50 and DL2000 plus DNA markers were purchased from Tsingke Biotechnology Co., Ltd. (Beijing, China). Fluorescent dyes were sourced from TransGen Biotechnology Co., Ltd. (Beijing, China), and kanamycin and crystal violet were obtained from Beyotime Biotechnology Co., Ltd. (Shanghai, China). Detailed information about the sequences of genes and primers used in this study are listed in Table 1. Gene-specific primers were designed based on the conserved sequences of the crp gene and promoter regions from *Klebsiella pneumoniae* genomes available in the NCBI GenBank database. Primer design was performed using oligo 7 software to ensure specificity and to incorporate the required restriction enzyme sites.

**Table 1:** Detailed information about the sequences of genes and primers used in this study.

Genes	Sequences (5′-3′)
P*tac*	GGATCCttgacaattaatcatcggctcgtataatgGAATTC^a^
*crp*-F/R	F: cgcGGATCCatgGTGCTTGGCAAACCGCAAA
	R: gggAAGCTTTTACTTGTCGTCATCGTCTTTGTA
P*kan*-F/R	F: gaAGATCTGAAGATCCTTTGATCTTTTC
	R: gcgGGATCCAACACCCCTTGTATTACT
P*tac*-F/R	F: cgcGGATCCttgacaattaat
	R: ggcGAATTCcattatacgagccg
16s-F/R	F: CATCATGGCCCTTACGACCAG
	R: ACGATTACTAGCGATTCCGACT
*crp*	1: CCGCAAACAGACCCTACCCT
	2: AGCCACGGAGCCTTTAACGA

a The underlined regions indicate restriction enzyme sites.

### Construction of the *crp* gene overexpression KP strain

#### Acquisition of the promoter and construction of the overexpression vector

The kanamycin resistance gene promoter (P*kan*) was amplified from pET-28a (+) plasmid using primers (Table 1) engineered with *BglII*/*BamHI* restriction sites. Polymerase chain reaction (PCR) was performed in a 25 μL reaction system containing 7.5 μL ddH₂O, 12.5 μL 2 × Rapid Taq Master Mix, 1 μL each primer, and 3 μL template. The cycling conditions were as follows: initial denaturation at 95 °C for 5 min, followed by 30 cycles of 95 °C for 15 s, 60 °C for 15 s, and 72 °C for 15 s per kb, ended with a final extension at 72 °C for 5 min. The amplified products were purified using a gel recovery kit and subjected to double digestion with *BglII*/*BamHI*, along with the pET-28a (+) plasmid, at 37 °C for 10 min in a 50 μL reaction system containing 5 μL 10 × QuickCut Green Buffer, 1 μL of each enzyme, 40.5 μL ddH₂O, and 2.5 μL DNA. After purification, the digested fragments were ligated at a 1:1 volume ratio in a 9.5 μL reaction system containing 4 μL each of fragment and vector, 1 μL 10 × T4 Ligase Buffer, and 0.5 μL T4 Ligase at 65 °C for 1 h. The ligation mixture was then transformed into DH5α via heat shock at 42 °C for 90 s. Positive clones (pE*kan*) were screened by colony PCR using primers P*kan*-F/R (Table 1) and verified by *BglII*/*BamHI* restriction digestion. The expected PCR product size was 130 base pairs (bp).

For construction of the pE*tac* vector, a synthetic P*tac* promoter fragment which was designed based on the sequence reported by Ma et al. (2013) and engineered with *BamHI* and *EcoRI* restriction sites at the 5′ and 3′ ends, respectively, was synthesized and sequence-verified by Sangon Biotech (Shanghai, China). Both the pET-28a vector and the P*tac* fragment were double-digested with *BamHI* and *EcoRI* under previously described conditions. The digested products were purified and ligated, and the resulting ligation mixture was transformed DH5α to generate the pE*tac* plasmid.

#### Cloning of the *crp* gene and construction of the recombinant plasmids pE*kan*-CRP and pE*tac*-CRP

**Figure 1:**
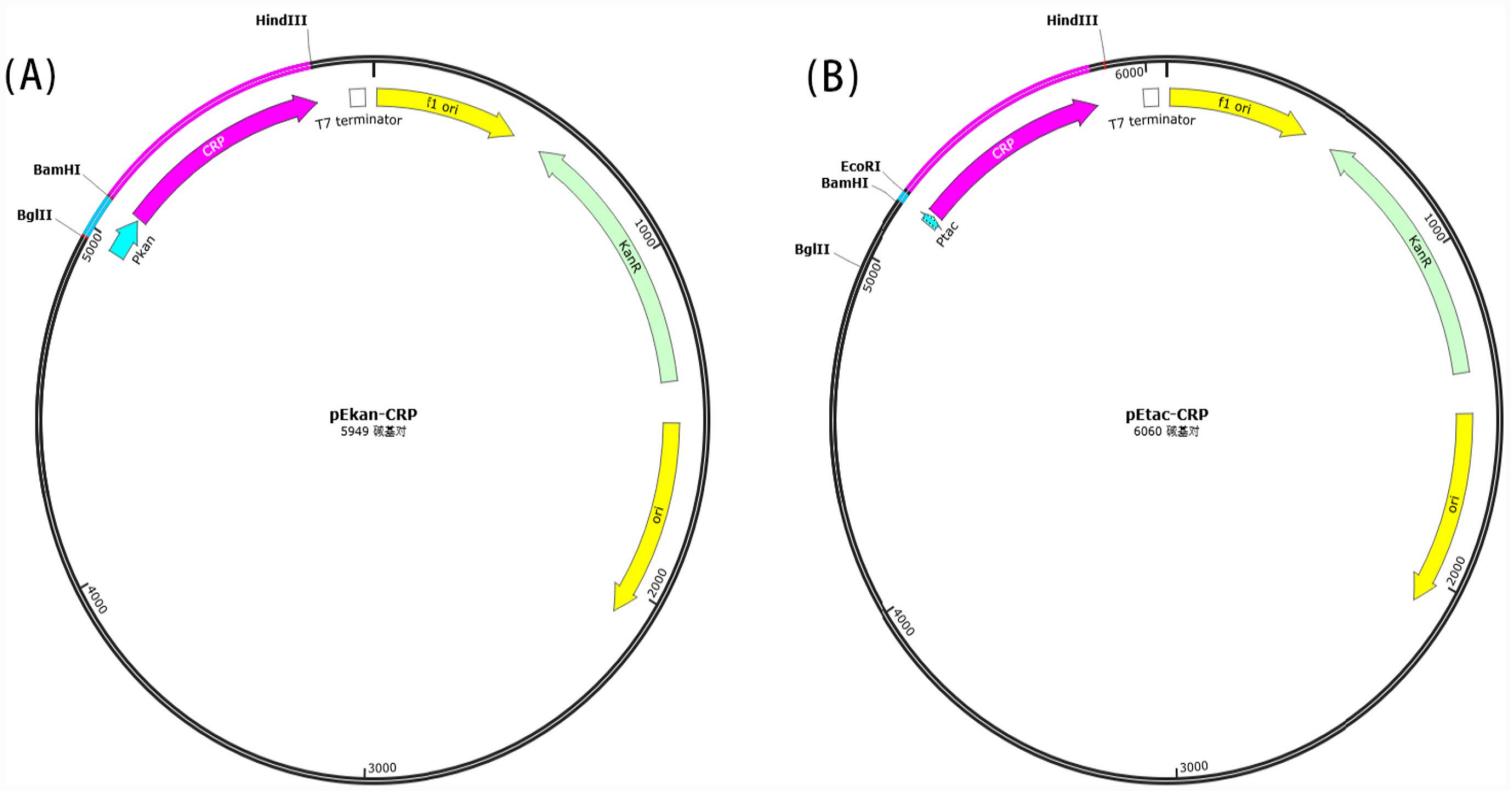
Sketch representations showing the plasmid maps of pE*kan*-CRP **(A)** and pE*tac*-CRP **(B)**.

Genomic DNA was extracted from the KP-L strain, and the *crp* gene was amplified using its primers *crp* (Table 1) engineered with *EcoRI* and *HindIII* restriction sites. The expected size of the PCR product was 643 bp. After gel purification, the amplified fragment and the pE*kan* plasmid were double-digested with *EcoRI* and *HindIII* under previously described conditions. The digested products were ligated at a 1:1 ratio to construct the recombinant plasmid pE*kan*-CRP. The ligation mixture was transformed into DH5α, and positive clones were identified by colony PCR with its primers *crp*-F/R (Table 1) and verified by double restriction enzyme digestion, with the expected PCR product size being 773 bp. The recombinant plasmid pE*tac*-CRP, places the *crp* gene under the control of the *tac* promoter, was commercially synthesized by Sangon Biotech according to the design of this study and delivered as a clonal stock in DH5α. The sequence of the entire expression cassette was fully verified. Figure 1 illustrates the plasmid maps of the pE*kan*-CRP and pE*tac*-CRP.

#### Electrotransformation of KP

Preparation of KP-L competent cells began by harvesting the cells during the logarithmic growth phase, followed by three washes with pre-chilled sterile water. The final cell pellet was resuspended in 200 μL of 10% glycerol. For transformation, 5 μL of each recombinant plasmid was added to the competent cells and incubated on ice for 5 min. Electroporation was performed using the following parameters: 2.5 kV, 200 *Ω*, 25 μF. Immediately after electroporation, 800 μL of antibiotic-free LB medium was added to the cells for recovery at 37 °C and 200 rpm for 5 h. The recovered culture was then plated on LB agar containing 50 μg/mL kanamycin and incubated at 37 °C for 16 h. Positive transformants were identified by PCR using the P*kan*-F and *crp*-R primers (Table 1).

#### Screening and identification of recombinant strains

Single, smooth, well-rounded colonies were selected and suspended in ultrapure water to provide templates for PCR amplification. Colony PCR was performed using the primers P*kan*-F and *crp*-R for pE*kan*-CRP, and PE*tac*-F and *crp*-R for pE*tac*-CRP, under the same reaction conditions and cycling program as previously described. The expected PCR product sizes were approximately 773 bp for pE*kan*-CRP and 669 bp for pE*tac*-CRP.

### Validation of *crp*-overexpression strain

#### Reverse transcription quantitative PCR analysis

Total RNA was extracted from the recombinant *Klebsiella pneumoniae* strain KP-L using the RNAprep Pure Cell/Bacteria Kit. RNA integrity and concentration were verified spectrophotometrically, followed by DNase I treatment to eliminate genomic DNA contamination. First-strand cDNA was synthesized from 1 μg of total RNA using the TransScript® One-Step gDNA Removal and cDNA Synthesis SuperMix, according to the manufacturer’s protocol.

Quantitative real-time PCR (qPCR) was performed using SYBR Green I chemistry on a Real-Time PCR system. The 20 μL reaction mixture contained SYBR Green mix, gene-specific primers for *crp(crp-1/2)*, and diluted cDNA template. The 16S rRNA gene served as an endogenous control. The thermal cycling conditions were: initial denaturation at 94 °C for 30 s, followed by 40 cycles of 94 °C for 5 s and 60 °C for 30 s. A melting curve analysis was included to confirm amplification specificity. All reactions were performed in triplicate.

#### Immunoblotting analysis

Five hundred microliters of bacterial culture were centrifuged, and the pellet was washed with PBS. Bacterial cells were lysed with B-PER™ Complete Bacterial Protein Extraction Reagent (Thermo Fisher, Cat# 89821) at a ratio of 5 mL per gram of cell wet weight at room temperature for 15 min. The supernatant was collected, mixed with 5 × loading buffer, and boiled for subsequent analysis. For each sample, 60 μg of protein was separated by SDS-PAGE using an 8–12% separating gel and a 5% stacking gel, with electrophoresis performed at 80 V for the separating gel and 60 V for the stacking gel. Proteins were transferred to a PVDF membrane using a wet transfer system at 100 V for 2 h. The membrane was blocked with 5% BSA in TBST for 1 h, then incubated overnight at 4 °C with primary antibodies (Anti-CRP, BioLegend Cat# 664304, diluted 1:500; Anti-rpoβ, Abcam Cat# ab191598, diluted 1:2000). After washing with TBST, the membrane was incubated with HRP-conjugated secondary antibodies (Goat anti-Mouse IgG, Thermo Pierce Cat# 31160, and/or Goat anti-Rabbit IgG, Thermo Pierce Cat# 31210, diluted 1:5000) for 1 h at room temperature. Detection was performed by treating the membrane with ECL substrate (SuperSignal® West Dura Extended Duration Substrate, Thermo Pierce Cat# 34075) for 1 min and exposing it to X-ray film for 5–10 min.

### Phenotypic differences between *crp*-overexpression and wild-type KP-L strains

#### Growth curve analysis

To assess the metabolic burden in the overexpression strain, pure cultures of wild-type (WT) and *crp*-overexpressing (OE) strains were inoculated into LB broth and incubated at 37 °C with shaking at 500 rpm. A 10 μL aliquot from each culture was transferred to fresh LB broth and incubated under the same conditions. At 1-h intervals, bacterial suspensions were homogenized and transferred to a 48-well plate for OD₆₀₀ measurement.

#### Analysis of drug resistance phenotype

The antimicrobial susceptibility of the WT and OE strains was determined using the Kirby-Bauer disk diffusion method following CLSI guidelines. Bacterial suspensions were adjusted to the optimal concentration and uniformly spread onto Mueller-Hinton (MH) agar plates. Twenty-three antibiotic disks representing seven major classes commonly used in clinical practice were placed on the agar surface. Plates were incubated upside down at 37 °C overnight.

#### Quantitative analysis of biofilm formation ability

Biofilm formation was assessed using a semi-quantitative crystal violet staining method in 96-well plates (Niemirowicz et al., 2016). Specifically, bacterial suspensions were adjusted to 1.5 × 10^8^ CFU/mL, and 10 μL of each suspension was added to wells containing 190 μL of LB broth. Each strain was tested in three technical replicates, with blank controls included. After 48 h of static incubation, biofilms were stained with crystal violet, washed with sterile phosphate buffered saline, and the dye was solubilized for OD570 measurement. The absorbance values from replicate wells within each experiment were averaged. The entire assay was performed in three independent experiments.

#### Statistical analysis

All quantitative data, including OD₆₀₀ values from growth curves, OD₅₉₀ values from biofilm assays, inhibition zone diameters, and relative gene/protein expression levels, are presented as mean ± standard error (SE) of at least three independent experiments. For biofilm assays, each biological replicate consisted of three technical replicates. For growth curves, measurements were taken from duplicate cultures in each independent experiment. For Reverse Transcription Quantitative PCR (RT-qPCR) analysis, the relative expression level of *crp* mRNA was calculated using the 2^−ΔΔCt^ method, with data presented as fold changes normalized to the WT group. The 16S rRNA gene served as an endogenous control. For Western blot analysis, band intensity was quantified using ImageJ software. The relative expression of CRP protein was calculated as the ratio of CRP band intensity to that of the internal control rpoβ. Statistical comparisons between WT OE groups were performed using Student’s *t*-test. A *p*-value of less than 0.05 was considered statistically significant. All statistical analyses were conducted using GraphPad Prism software (version 10.1.2).

## Results

### Successful cloning of the constitutive promoter and *crp* gene

**Figure 2:**
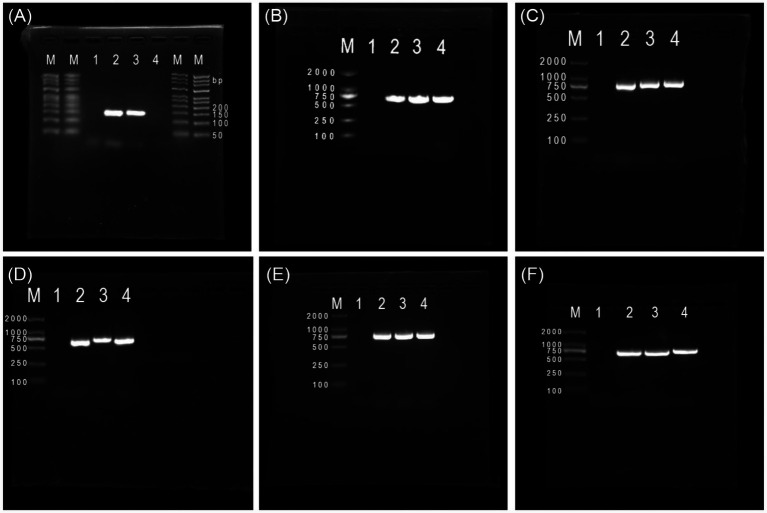
Gel electrophoresis images of the cloned genes and constructed recombinant strain. **(A)** Gel electrophoresis image of the cloned *Pkan* gene: M, DNA marker DL50; lanes 1 and 4, blank controls; lanes 2 and 3, *Pkan* gene; **(B)** gel electrophoresis image of the cloned *crp* gene: M, DNA marker DL2000; lanes 1 and 4, blank controls; lanes 2 and 3, *crp* gene; **(C)** gel electrophoresis image of the constructed *pEkan-CRP* recombinant plasmid: M, DNA marker DL2000; lanes 1 and 4, blank controls; lanes 2 and 3, *pEkan-CRP* recombinant plasmid. **(D)** Gel electrophoresis image of the constructed *pEtac-CRP* recombinant plasmid: M, DNA marker DL2000; lanes 1 and 4, blank controls; lanes 2 and 3, *pEtac-CRP* recombinant plasmid. **(E,F)** Gel electrophoresis image of the constructed *crp*-overexpression KP-L strain: M, DNA marker DL2000; lanes 1 and 4, blank controls; lanes 2 and 3, *pEkan-crp* **(E)** and *pEtac-crp* **(F)**.

PCR amplification of the P*kan* promoter from pET28a plasmid yielded a product of approximately 130 bp (Figure 2A). Amplification of the *crp* gene from KP-L genomic DNA produced a fragment of approximately 642 bp (Figure 2B).

### Construction and verification of the expression vector and recombinant KP-L strain

Colony PCR of transformed DH5α clones showed bands of expected sizes for pE*kan*-CRP (773 bp) and pE*tac*-CRP (669 bp) (Figures 2C,D). Electroporation of pE*kan*-CRP and pE*tac*-CRP into KP-L yielded transformants confirmed by colony PCR with expected band sizes (Figures 2E,F).

### CRP expression levels in WT and OE KP-L strains

**Figure 3:**
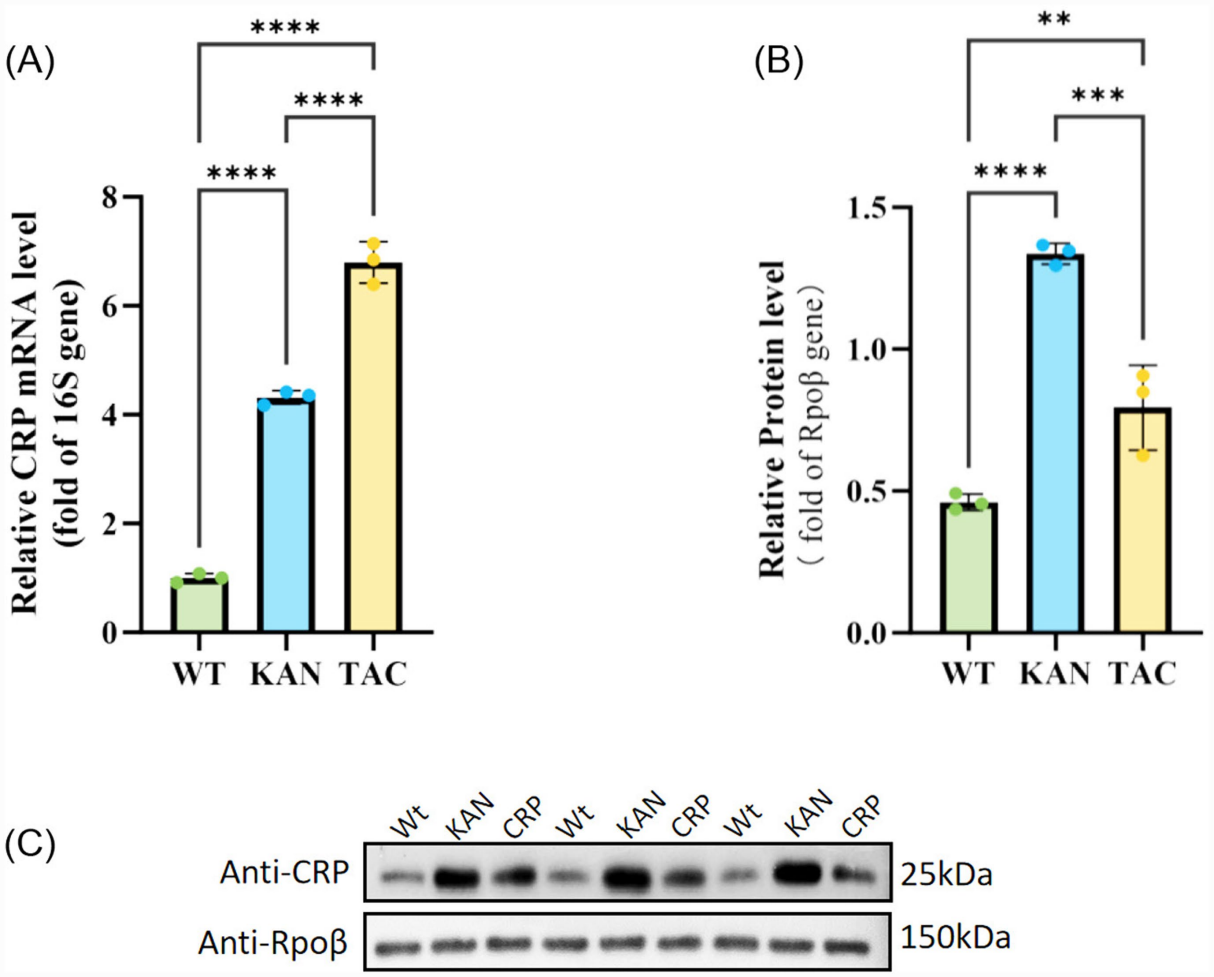
Expression level of *crp* gene and protein in wild-type and *crp*-overexpression KP-L strains. **(A,B)** Bar plots showing the relative expression level of CRP in the wild-type and *crp*-overexpression KP-L strains at both gene **(A)** and protein **(B)** levels; **(C)** Representative immunoblot pictures of CRP protein in the wild-type and *crp*-overexpression KP-L strains. In **(A,B)**, data are expressed as mean ± standard error and statistically analyzed using Student’s *t*-test: **p < 0.01; ***p < 0.001; ****p < 0.0001.

RT-qPCR analysis showed significantly higher *crp* transcript levels in KAN (4.318 ± 0.124) and TAC (6.797 ± 0.378) groups compared to WT (1.00 ± 0.083) (*p* < 0.01) (Figure 3A), Western blot analysis confirmed elevated CRP protein levels in KAN (1.337 ± 0.036) and TAC (0.794 ± 0.142) groups versus WT (0.435 ± 0.019) (*p* < 0.05) (Figures 3B,C).

### Growth curves of the WT and OE KP-L strains

**Figure 4:**
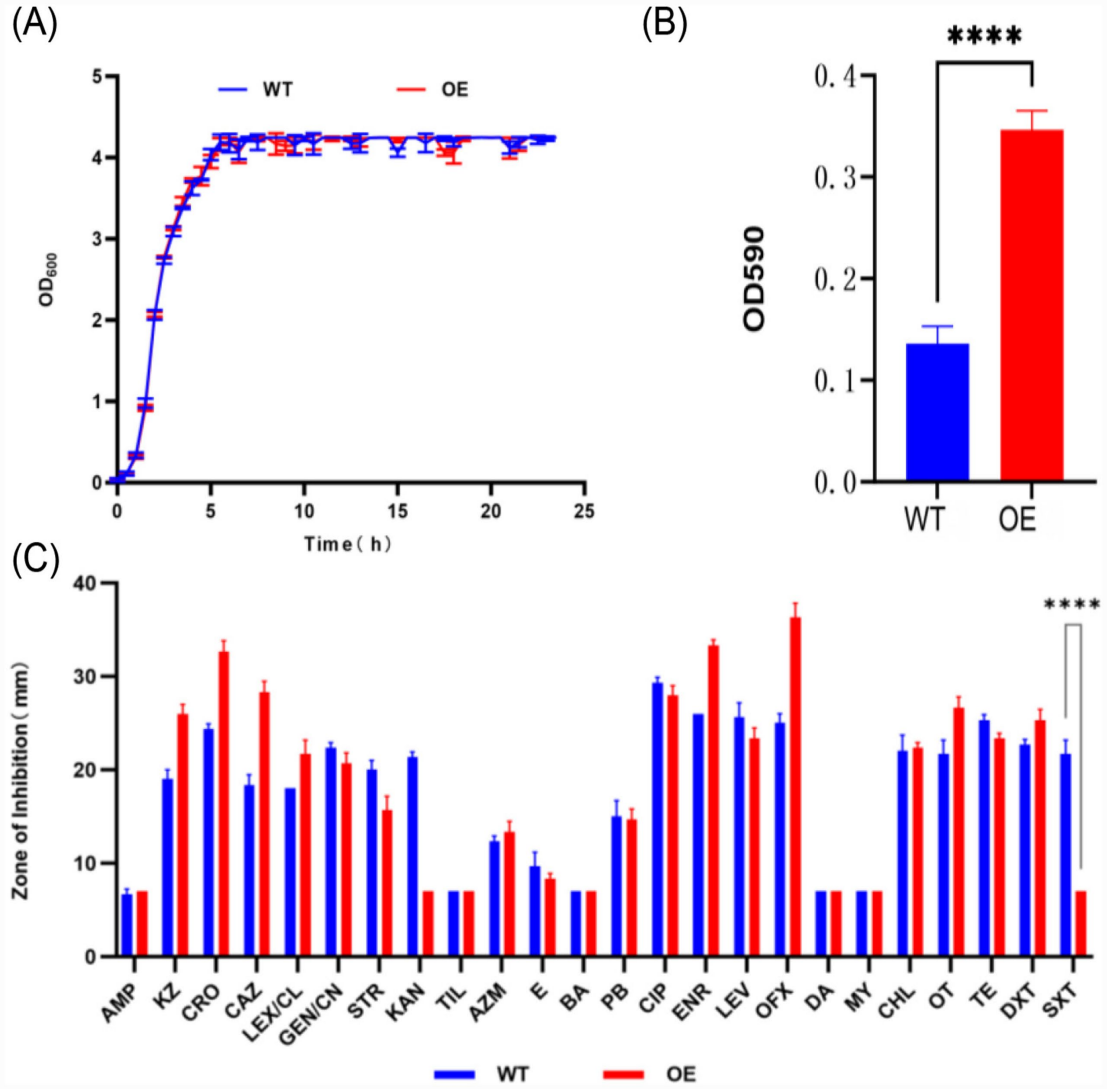
Phenotypes of the wild-type and *crp*-overexpression KP-L strains. **(A)** Dot-line plot showing the growth curves of the wild-type (WT) and *crp*-overexpressing (OE) KP-L strains; **(B)** Bar plot displaying the OD590 absorbance values of dyed biofilm in the WT and OE KP-L strains; **(C)** Grouped-bar plot demonstrating the inhibition zone diameters of the WT and OE KP-L strains in the zone of inhibition assay against multiple antibiotics. AMP, ampicillin; KZ, cefazolin; CRO, ceftriaxone; CAZ, ceftazidime; LEX/CL, cephalexin/clavulanate; GEN/CN, gentamicin; STR, streptomycin; KAN, kanamycin; TIL, tilmicosin; AZM, azithromycin; E, erythromycin; BA, bacitracin; PB, polymyxin B; CIP, ciprofloxacin; ENR, enrofloxacin; LEV, levofloxacin; OFX, ofloxacin; DA, clindamycin; MY, lincomycin; CHL, chloramphenicol; OT, oxytetracycline; TE, tetracycline; DXT, doxycycline; SXT, co-trimoxazole. Data are expressed as mean ± standard error and statistically analyzed using Student’s *t*-test **(B,C)**: ****p < 0.0001.

Kinetic monitoring of OD_600_ values for WT and OE KP-L strains over 24 h of shaking incubation at 37 °C demonstrated that both strains exhibited typical growth curve characteristics, with a brief lag phase (0–1.5 h) and no significant difference in initial biomass (WT initial OD_600_: 0.034–0.056; OE: 0.032–0.059). During the exponential phase (1.5–7 h), OD_600_ values increased rapidly in both strains, and biomass remained highly consistent at time points (e.g., at 5 h: WT 2.016–2.133, OE 2.035–2.098; at 7 h: WT 3.031–3.141, OE: 3.111–3.157). In the stationary phase (>7 h), both strains reached similar plateau biomass (approximate 4.0–4.2) by 16 h, which was maintained through the 24-h period (WT: 4.248 ± 0.002; OE: 4.248 ± 0.002) (Figure 4A).

### Biofilm formation capacity of the WT and OE KP-L strains

Crystal violet staining revealed significantly higher OD590 values in the OE group (0.347 ± 0.129) compared to WT (0.136 ± 0.120) (*p* < 0.05) (Figure 4B).

### Drug resistance of the WT and OE KP-L strains

Disk diffusion assays showed similar resistance profiles for most antibiotics in WT and OE strains. A notable difference was observed for co-trimoxazole (SXT): WT was susceptible (inhibition zone: 21.67 ± 1.53 mm), while OE was resistant (inhibition zone: 7 ± 0 mm) (Table 2; Figure 4C).

**Table 2:** Detailed information about the results of the zone of inhibition assay.

Antibiotic	Content	Resistance phenotype			IZD^b^ OE^c^ (mm)	RP^d^ OE	IZD^e^ WT (mm)	RP WT
		R^a^	I^a^	S^a^				
AMP	10	≤11	12–14	≥15	7	R	7	R
KZ	30	≤14	15–17	≥18	26	S	19	S
CRO	30	≤13	14–20	≥21	33	S	24	S
CAZ	30	≤14	15–17	≥18	29	S	19	S
LEX/CL	30	≤14	15–17	≥18	22	S	18	S
GEN/CN	10	≤10	11–14	≥15	20	S	22	S
STR	10	≤10	11–14	≥15	16	S	20	S
KAN	30	≤13	14–17	≥18	7	R	21	S
TIL	15	≤10	11–14	≥15	7	R	7	R
AZM	15	≤13	14–17	≥18	13	R	12	R
E	15	≤13	14–17	≥18	9	R	10	R
BA	10	≤13	–	≥14	7	R	7	R
PB	300	≤11	12–14	≥15	15	S	15	S
CIP	5	≤15	16–20	≥21	28	S	29	S
ENR	5	≤15	16–20	≥21	33	S	26	S
LEV	5	≤26	–	≥27	23	R	26	R
OFX	5	≤12	13–15	≥16	36	S	25	S
DA	2	≤14	15–20	≥21	7	R	7	R
MY	2	≤14	15–20	≥21	7	R	7	R
CHL	30	≤12	13–17	≥18	22	S	21	S
OT	30	≤18	19–21	≥22	26	S	22	S
TE	30	≤14	15–18	≥19	23	S	25	S
DXT	30	≤10	11–13	≥14	26	S	23	S
SXT	25	≤10	11–15	≥16	7	R	22	S

a R, resistant; I, intermediate; S, susceptible.

b IZD, inhibition zone diameter.

c OE, crp-overexpression KP-L strain.

d RP, resistance phenotype.

e IZD, inhibition zone diameter.

## Discussion

Our findings demonstrated that CRP overexpression in bovine-derived KP not only significantly enhances biofilm formation but also confers complete resistance to co-trimoxazole (Figure 4C). To our knowledge, this study is the first to successfully construct a *crp*-overexpression model in a bovine isolate using both a constitutive promoter (P*kan*) and an inducible promoter (P*tac*). Molecular and phenotypic analyses systematically revealed the central role of *crp* gene in regulating biofilm architecture and antibiotic resistance. Quantitative assays clearly showed a highly significant increase in biofilm biomass in the OE strain compared to the WT strain (Figure 4C), directly confirming CRP’s crucial regulator role in the biofilm development of KP. Similarly, *crp* deletion mutants (Δ*CRP*) in *E. coli* was found to exhibit substantial biofilm formation defects, confirming CRP’s positive regulatory function in this process (Hufnagel et al., 2016). This aligns with its known function in *K. pneumoniae*, where the cAMP-CRP complex positively regulates the *mrk* operon, essential for type III fimbriae synthesis which facilitates surface attachment and biofilm maturation (Panjaitan et al., 2019). It must be note that the regulatory strength of CRP on biofilm formation in bovine-derived strains may differ from that in human clinical isolates (e.g., NTUH-K2044) due to host adaptive evolution, and further comparative genomics and transcriptomics are warranted to clarify this potential host specificity.

A key finding of this study is the significant transcriptional-translational decoupling in *crp* gene regulation warrants discussion. Although the inducible promoter P*tac* drove higher transcriptional levels of the *crp* gene compared to the constitutive promoter P*kan* (Figure 3A), immunoblotting analysis revealed that the CRP protein expression level was significantly higher in the P*kan* group than in the P*tac* group (Figure 3B). This discrepancy may stem from post-transcriptional bottlenecks under strong induction, such as translational inhibition, protein misfolding leading to degradation, or activation of stress-responsive proteases. This observation underscores the importance of validating phenotypic studies at the protein level and justified our functional analysis using the KAN (Pkan-driven) strain: it maintains high transcriptional levels while achieving stable and detectable protein expression, effectively avoiding non-specific stress interference associated from extreme induction. Additionally, CRP overexpression was not found to impose any observable metabolic burden on bacterial proliferative capacity. Growth kinetic analysis indicated no significant differences in the duration of the lag phase, specific growth rate, or maximum biomass yield between the OE and WT strains (Figure 4A), demonstrating that CRP-mediated resistance enhancement is not a side effect of growth inhibition but a direct result of activating its specific regulatory network. This feature supports the feasibility of intervention strategies targeting the *crp* gene.

Notably, we also observed that *crp*-overexpression resulted in a marked reversal of the resistance phenotype to cotrimoxazole (Figure 4C). We hypothesize that this resistance reversal likely results from CRP’s multifaceted regulatory mechanisms: it may directly or indirectly activate multidrug efflux pump genes such as *acrAB-tolC* (Ruiz and Levy, 2010), enhancing the active efflux of sulfamethoxazole; concurrently, CRP-driven metabolic reprogramming may upregulate endogenous folate synthesis and uptake-related pathways (e.g., *folA*, *folP*) (Sköld, 2000), compensating for the competitive inhibition of dihydrofolate reductase by cotrimoxazole and ultimately remodeling the resistance phenotype. However, it is crucial to note that these mechanistic insights remain speculative within the scope of the present work. Our study primarily establishes the phenotypic link but does not include direct molecular validation. To conclusively elucidate the mechanism, future investigations are warranted. These should include: (i) constructing isogenic crp knockout mutants to confirm the necessity of CRP for both biofilm formation and cotrimoxazole resistance; (ii) quantifying the minimum inhibitory concentration (MIC) of cotrimoxazole to precisely define the resistance level; (iii) profiling the expression of efflux pump genes (e.g., acrAB) and folate pathway genes; and (iv) measuring intracellular sulfamethoxazole accumulation to directly assess efflux pump activity. Addressing these points will bridge the gap between phenotypic observation and molecular mechanism.

## Conclusion

In conclusion, this study systematically investigated the role of the global regulator CRP in a bovine-derived *K. pneumoniae* isolate through a gain of function approach. We successfully constructed and validated CRP-overexpressing strains using both constitutive and inducible promoters, and comprehensively evaluated their phenotypes compared to the wild-type strain. Our principal findings are threefold: First, CRP functions as a key positive regulator of biofilm formation; Second, CRP overexpression remodels the antibiotic susceptibility to resistance specifically to cotrimoxazole and kanamycin, with the shift for cotrimoxazole being the most profound. Third, this CRP mediated phenotypic shift occurs without imposing a significant metabolic burden on bacterial growth, and we observed a notable decoupling between crp transcript levels and CRP protein abundance under strong induction. These results establish a direct link between CRP expression levels, biofilm architecture, and specific antibiotic resistance in a bovine pathogen. They suggest that targeting the CRP regulatory network could be a novel strategy to counteract biofilm-associated resistance. Future research should focus on elucidating the precise molecular mechanisms (such as identifying CRP’s direct transcriptional targets related to cotrimoxazole resistance) and comparing CRP’s regulon across hosts to understand its adaptive evolution.

## Data Availability

The original contributions presented in the study are included in the article/Supplementary material, further inquiries can be directed to the corresponding author.
