# Supplementary figures and images for: Overexpression of the crp gene promotes biofilm formation and increases antibiotic resistance in bovine-derived Klebsiella pneumoniae
Source: Front Microbiol. 2026 Jan 26;17:1766955. doi: 10.3389/fmicb.2026.1766955 (PMC12883750; doi:10.3389/fmicb.2026.1766955)

| Group | OE | | | WT | | |
| --- | --- | --- | --- | --- | --- | --- |
| PB  AMP  LEV  E | 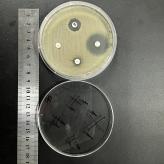 | 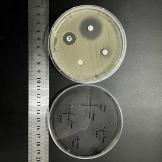 | 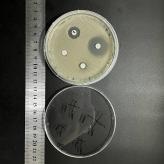 | 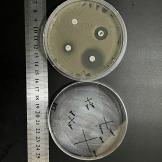 | 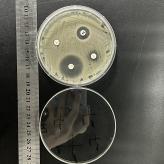 | 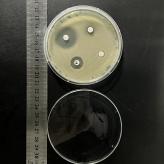 |
| DXT  S  SXT  CIP  CRO | 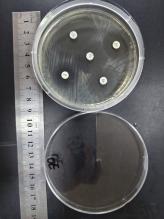 | 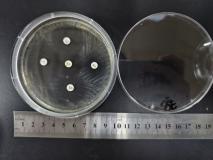 | 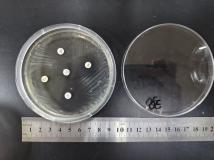 | 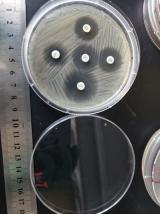 | 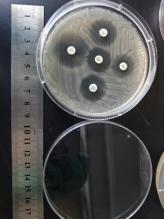 | 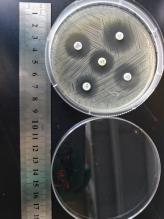 |
| OFX  K  TIL  MY  ENR | 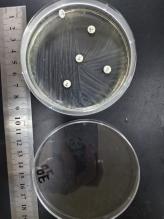 | 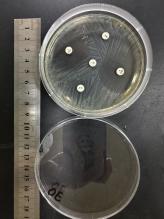 | 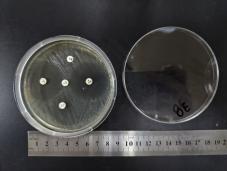 | 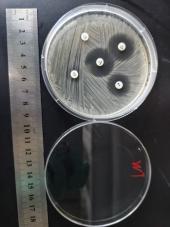 | 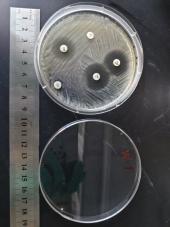 | 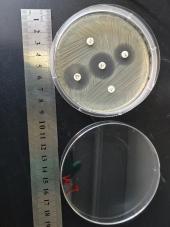 |
| BA  OT  CAZ  DA  CL | 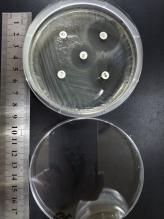 | 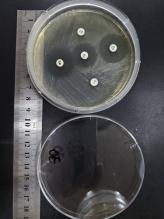 | 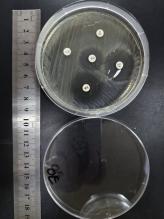 | 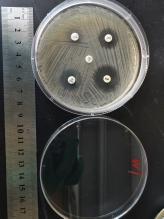 | 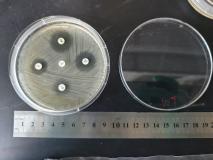 |  |
| TE  KZ  AZM  CN  C | 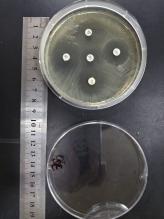 | 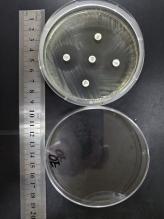 | 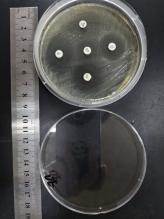 | 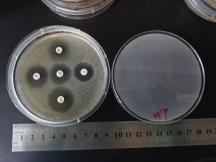 | 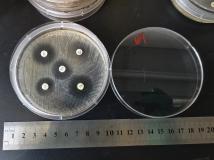 | 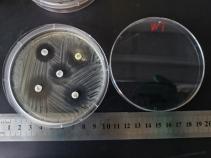 |
|  |  |  |  |  |  |  |
|  |  |  |  |  |  |  |

Supplement: Supplementary file 1 [file Table_1.DOCX]
